# Supplementary material for: Physical Activity Energy Expenditure Predicts Quality of Life in Ambulatory School-Age Children with Cerebral Palsy
Source: J Clin Med. 2022 Jun 11;11(12):3362. doi: 10.3390/jcm11123362 (PMC9225112; doi:10.3390/jcm11123362)
Supplement: Supplementary file 1 [file jcm-11-03362-s001.zip › jcm-1731359-supplementary.pdf]

Table S1. Hierarchical regression analysis results.

	Dependent variable										
	PedsQL(parent) - Physical domain		PedsQL(parent) - emotional domain		PedsQL(parent) - social domain		PedsQL(parent) - school domain		PedsQL(parent) - total score		
	B	beta	B	beta	B	beta	B	beta	B	beta	VIF
Controlled variables											
Unilateral CP	-6.016	-.125	1.318	.051	-14.592	-.354	-6.261	-.173	-6.339	-.227	1.943
Girls	6.040	.127	-1.385	-.054	**14.388***	**.354***	10.639	.298	7.240	.263	1.163
GMFCS level_II	-31.256	-.647	-1.943	-.075	**-23.024***	**-.558***	-9.849	-.272	**-18.440***	**-.660***	2.276
GMFCS level_III	-45.935	-.650	-9.380	-.249	-21.666	-.359	2.116	.040	**-22.267***	**-.545***	2.293
Overweight	.464	.007	-3.919	-.104	-.084	-.001	-6.909	-.130	-2.211	-.054	1.519
Independent variables											
PAEE	1.899	.108	-1.774	-.188	-1.334	-.089	3.459	.261	.737	.072	3.386
%MVPA	-.720	-.067	-.835	-.146	2.297	.252	-2.211	-.276	-.413	-.067	3.588
Activity Counts(counts/min)	-.002	-.018	.006	.126	.005	.056	.018	.252	.006	.104	3.502
R^2	.482		.093		.333		.187		.442		-

VIF: Variance Inflation factor, CP: cerebral palsy, PedsQL: Pediatric Quality of Life inventory, GMFCS: Gross Motor Function Classification System, PAEE: Physical Activity Energy Expenditure, LPA: Light Physical Activity, MVPA: Moderate to Vigorous Physical Activity

* P < 0.05

B: unstandardized coefficient

beta: standardized coefficient

Table S2. Hierarchical regression analysis results.

	Dependent variable				
	CHQ-PF50- Physical		CHQ-PF50-Psychosocial		
	B	beta	B	beta	VIF
Controlled variables					
Unilateral CP	-3.166	-.127	-3.241	-.192	1.943
Girls	3.100	.126	-.589	-.035	1.163
GMFCS level_II	-10.709*	-.429*	-3.865	-.229	2.276
GMFCS level_III	-24.943*	-.683*	-1.153	-.047	2.293
Overweight	-2.949	-.081	-4.197	-.170	1.519
Independent variables					
PAEE	2.438	.268	1.443	.235	3.386
%MVPA	-.487	-.088	-1.000	-.268	3.588
Activity Counts(counts/min)	.002	.043	.001	.019	3.502
R^2	.593		.078		-

VIF: Variance Inflation factor, CP: cerebral palsy, CHQ-PF50: Child Health Questionnaire – Parent form 50, GMFCS: Gross Motor Function Classification System, PAEE: Physical Activity Energy Expenditure, LPA: Light Physical Activity, MVPA: Moderate to Vigorous Physical Activity
* P < 0.05
B: unstandardized coefficient
beta: standardized coefficient
